# Comparison of Longitudinal *In Vivo* Measurements of Retinal Nerve Fiber Layer Thickness and Retinal Ganglion Cell Density after Optic Nerve Transection in Rat

**DOI:** 10.1371/journal.pone.0113011

**Published:** 2014-11-13

**Authors:** Tiffany E. Choe, Carla J. Abbott, Chelsea Piper, Lin Wang, Brad Fortune

**Affiliations:** Discoveries in Sight Research Laboratories, Devers Eye Institute and Legacy Research Institute, Legacy Health, Portland, Oregon, United States of America; Hanson Institute, Australia

## Abstract

**Purpose:**

To determine the relationship between longitudinal *in vivo* measurements of retinal nerve fiber layer thickness (RNFLT) and retinal ganglion cell (RGC) density after unilateral optic nerve transection (ONT).

**Methods:**

Nineteen adult Brown-Norway rats were studied; N = 10 ONT plus RGC label, N = 3 ONT plus vehicle only (sans label), N = 6 sham ONT plus RGC label. RNFLT was measured by spectral domain optical coherence tomography (SD-OCT) at baseline then weekly for 1 month. RGCs were labeled by retrograde transport of fluorescently conjugated cholera toxin B (CTB) from the superior colliculus 48 hours prior to ONT or sham surgery. RGC density measurements were obtained by confocal scanning laser ophthalmoscopy (CSLO) at baseline and weekly for 1 month. RGC density and reactivity of microglia (anti-Iba1) and astrocytes (anti-GFAP) were determined from post mortem fluorescence microscopy of whole-mount retinae.

**Results:**

RNFLT decreased after ONT by 17% (p<0.05), 30% (p<0.0001) and 36% (p<0.0001) at weeks 2, 3 and 4. RGC density decreased after ONT by 18%, 69%, 85% and 92% at weeks 1, 2, 3 and 4 (p<0.0001 each). RGC density measured *in vivo* at week 4 and *post mortem* by microscopy were strongly correlated (R = 0.91, p<0.0001). *In vivo* measures of RNFLT and RGC density were strongly correlated (R = 0.81, p<0.0001). In ONT- CTB labeled fellow eyes, RNFLT increased by 18%, 52% and 36% at weeks 2, 3 and 4 (p<0.0001), but did not change in fellow ONT-eyes sans CTB. Microgliosis was evident in the RNFL of the ONT-CTB fellow eyes, exceeding that observed in other fellow eyes.

**Conclusions:**

*In vivo* measurements of RNFLT and RGC density are strongly correlated and can be used to monitor longitudinal changes after optic nerve injury. The strong fellow eye effect observed in eyes contralateral to ONT, only in the presence of CTB label, consisted of a dramatic increase in RNFLT associated with retinal microgliosis.

## Introduction

Glaucoma is the most common optic neuropathy and is the second leading cause of blindness worldwide [1]. Though intraocular pressure (IOP) is the most important treatable risk factor and currently the only target for treatment, the mechanisms by which IOP ultimately damages optic nerve axons remain unclear [2], [3]. Thus, experimental models of glaucoma are critical for elucidating details of pathophysiological mechanisms as well as for testing new avenues of therapy. Experimental models of glaucoma are commonly based on elevated IOP, often induced unilaterally, for example in non-human primates or rodents; or in the case of heritable models such as the DBA/2J mouse, IOP becomes chronically elevated in both eyes during the course of aging [4]–[9]. Common outcome measures for experimental glaucoma models include anatomical counts of retinal ganglion cell (RGC) soma and/or orbital optic nerve axons, which require sacrifice of the animal for histological processing. The ability to visualize RGCs *in vivo* has raised the possibility of longitudinal evaluation within animals, which could help to both reduce the number of animals required to adequately power a scientific study (instead of sacrificing a different set of animals at each time point) and to minimize the possibility of errors arising when inferences about longitudinal time course and inter-relationships are drawn from cross-sectional data [10]–[13].

Various techniques for visualizing RGCs *in vivo* have evolved over the past two decades but generally involve imaging by either epifluorescence microscopy [12], [13], fundus photography [14] or by confocal scanning laser microscopy (CSLM)[10], [15], [16] or ophthalmoscopy (CSLO)[17]–[27] after introduction of a fluorescent tracer via retrograde transport from the midbrain [10]–[12], [14], [16], [18], [22], [26], [27] or a fluorescent reporter molecule whose expression is driven by a promoter that is relatively specific to RGCs [14], [15], [19]–[21], [23]–[25]. In some transgenic lines such as the Thy-1 YFP mouse, expression is limited to a small enough proportion of RGCs to enable visualization *in vivo* of even fine dendritic structure by CSLO [15], [21]. Alternatively, the addition of adaptive optics (AO) to CSLO has also enabled visualization *in vivo* of RGC fine dendritic structure [22]–[24]. More recently, imaging of RGC light responses and even finer structure in the living mouse eye have been achieved using AO-CSLO with and without two-photon excitation capability [28], [29]. By longitudinal imaging *in vivo*, several of these previous studies have demonstrated decline in RGC density, changes in RGC soma size, dendritic arbor and axonal integrity, or RGC apoptosis after optic nerve injury [10]–[14], [17], [18], [20], [21], [25], [27].

Similarly, the advancement of optical coherence tomography (OCT)[30] has enabled investigators to monitor longitudinal change in the structural characteristics of the optic nerve head (ONH), macula and retinal nerve fiber layer thickness (RNFLT) *in vivo*, for both clinical management of glaucoma [31]–[34] and evaluation of experimental models in a laboratory setting [25], [35]–[39]. Currently, there is no clinically approved method for labeling RGCs *in vivo* in humans. Hence, improving the understanding of the longitudinal relationship between *in vivo* RGC density and *in vivo* RNFLT in animal models of optic nerve injury is important for interpreting what RNFLT loss means for RGC loss in the clinical setting. No study has yet followed change of both RGC density and RNFLT within the same eyes longitudinally after optic nerve injury. Chauhan and colleagues [25] followed both RNFLT by spectral domain OCT (SD-OCT) and RGC density by CSLO in transgenic (Thy-1/CFP) mice after optic nerve transection, but these two measurements were performed in separate groups of mice and on different post injury time points and were not directly compared. We have developed methods for *in vivo* assay of axonal transport in RGCs based on CSLO imaging of a fluorescent tracer (cholera toxin beta subunit, CTB) and have begun to apply the techniques in conjunction with SD-OCT imaging to study experimental models of optic nerve injury [26], [39]. Though we noted during pilot work that the persistence of RGC label was sufficiently long in naïve eyes to potentially allow longitudinal evaluation of RGC density, we did not examine changes in labeled RGCs over time after experimental optic nerve injury [26]. Therefore, the purpose of this study was to determine the relationship between *in vivo* measurements of RGC density and RNFLT by longitudinal imaging of the same animals after unilateral optic nerve transection.

## Methods

### Subjects

Nineteen male Brown-Norway rats (*Rattus norvegicus*, Charles River Laboratories Inc., Willmington, MA) were used in this study. They were housed in pairs or groups of three under a 12-hour light/12-hour dark cycle with normal rat chow and water available ad libitum. At the start of the study the rats were 8–9 weeks in age and ranged 176–200 grams in weight. All experimental methods and animal care procedures conformed to the ARVO Statement for the Use of Animals in Ophthalmic and Vision Research, were carried out in strict accordance with the recommendations in the Guide for the Care and Use of Laboratory Animals of the National Institutes of Health and were approved (protocol permit #12-05) and monitored by the Institutional Animal Care and Use Committee (IACUC) at Legacy Health (USDA license 92-R-0002 and OLAW assurance A3234-01). All efforts were made to minimize suffering.

### Anesthesia

For imaging and optic nerve transection procedures, rats were anesthetized with an intramuscular injection combination of ketamine (55 mg/kg, Ketaset, Fort Dodge Animal Health, Fort Dodge, IA), xylazine (5 mg/kg, AnaSed, Lloyd, Inc., Shenandoah, IA) and acepromazine maleate (1 mg/kg, Vedco, Inc., St Joseph, MO). For stereotactic injections, animals were anesthetized using 2% isofluorane gas in 2∶1 N_2_O∶O_2_.

### Experimental Design

All nineteen rats underwent baseline OCT imaging *in vivo* to measure RNFLT bilaterally prior to any other procedure (Table 1). The two baseline imaging sessions were separated by approximately 1-week. Each rat was then allocated to one of three experimental groups. Group-1 rats (N = 10) had bilateral stereotactic injections of CTB into the superior colliculus in order to label RGCs by retrograde transport in both eyes, followed 48 hrs later by unilateral optic nerve transection in the right eye. Group-2 rats (N = 3) had a unilateral optic nerve transection on the right eye but the bilateral stereotactic injections into the superior colliculus contained only vehicle (no CTB). Group-3 rats (N = 6) had bilateral stereotactic injections of CTB into the superior colliculus, followed 48 hrs later by a sham optic nerve transection procedure. Methods are further detailed below in subsections.

**Table 1:** Timeline of procedures, overview of experimental design.

Timeline	Procedure
1 and 2 weeks prior to Day 0	Two, weekly baseline measurements of peripapillary RNFLT using SD-OCT.
Day 0	Stereotactic injections of CTB bilaterally into superior colliculus to label RGCs in Group-1 (ONT, N = 10) and Group-3 (ONT sham, N = 6), or vehicle in Group-2 (ONT without CTB control group, N = 3).
Day 2	Bilateral ocular fundus imaging by CSLO to determine “baseline” density of CTB-labeled RGCs, followed immediately by unilateral ONT or sham surgery.
Days 9–30	Weekly follow-up imaging *in vivo* (SD-OCT measurements of RNFLT and CSLO measurements of RGC density) for 4 weeks.
Day 30	Animals sacrificed, tissue preservation and harvest for histopathology.
Day 30+	Immunohistochemisty: retinas stained with anti-Iba1 and anti-GFAP, then whole-mounted for microscopy to evaluate reactivity of microglia and astrocytes, respectively. Epifluorescence microscopy measurements (ImageJ) of CTB-labeled RGC and Iba-1 labeled microglial cell densities.

### *In Vivo* SD-OCT Imaging Protocol

Longitudinal measurements of peripapillary RNFLT were acquired *in vivo* by SD-OCT (Spectralis OCT+HRA, Heidelberg Engineering GmbH, Heidelberg, Germany) as previously described [39], [40]. After induction of general anesthesia, rats were placed on a custom-built imaging stage and kept warm with a thermostatically-controlled system (TP650, Gaymar Industries, Inc., Orchard Park, NY). Pupils were dilated by topical administration of tropicamide (0.5%, Alcon Laboratories Inc., Fort Worth, TX) and phenylephrine (2.5%, Bausch and Lomb Pharmaceuticals Inc., Tampa, FL) and proparacaine hydrochloride (0.5%, Alcon) was applied for topical anesthesia. Custom rigid gas permeable contact lenses (3.5 mm posterior radius of curvature, 5.0 mm optical zone diameter, +5.0 diopter back vertex power) were then placed on both eyes to maintain corneal hydration and clarity. SDOCT scans consisted of 1536 A-scans equally spaced along a 12° diameter circular B-scan path. Digital transverse resolution in the rat eye is 1.6 µm/pixel (as determined empirically in our laboratory [26], [39], which matches exactly the theoretical derivation from a schematic eye model developed by Lozano and Twa [41]), axial resolution is 3.9 µm/pixel. The instrument's real-time eye tracking function was used to reduce speckle noise by averaging 100 sweeps for each B-scan recorded as well as to scan the identical location during all longitudinal follow-up sessions. SD-OCT raw data were exported for image segmentation and derivation of RNFLT measurements using custom software [39], [40]. Total retinal thickness (RT) was also measured from the same SD-OCT B-scan images as the distance between the segmentations defining the inner limiting membrane (ILM) and the Bruch's membrane/retinal pigment epithelium (RPE) complex.

RNFLT was measured in both eyes of each rat twice during baseline (prior to any other procedure such as stereotactic surgery, ONT or sham ONT surgery) and then repeated at weekly intervals following ONT (Groups 1 and 2), or sham ONT surgery (Group 3) for a total of 4-weeks post-injury follow-up (see Table 1).

### Retrograde RGC Labeling and Quantification *In Vivo*

RGCs in both eyes of each rat (Groups 1 and 3, N = 16 total) were labeled by retrograde transport of the fluorescent tracer CTB conjugated to AlexaFluor 488. Bilateral stereotactic 2 µl injections of 1% CTB dissolved in sterile PBS were made into the superior colliculus as previously described in detail [26], [39]. The stereotactic co-ordinates used were: −5.5 mm anterior-posterior, ±1.25 mm medial-lateral (both relative to the Bregma skull landmark), and −4.5, −4.25, −4.0 and −3.75 mm dorsal ventral (DV; from skull surface). At each DV location 0.5 µl of CTB was injected to optimize diffusion of CTB throughout each hemisphere of the superior colliculus. Buprenorphine analgesic was administered (0.075 mg/kg, IM) after completion of stereotactic surgery. In Group-2 rats (ONT+vehicle controls, N = 3), the stereotactic procedure was identical except that CTB was omitted so the injection contained only vehicle (sterile PBS, 2 µl total each hemisphere).

*In vivo* quantification of CTB-labeled RGC density was performed from CSLO (Spectralis OCT+HRA) images using ImageJ software as previously described in detail [26], [39]. CSLO images were centered on the optic disc and had a total area of 4.85 mm^2^ (4.67±0.04 mm^2^ after excluding the optic disc area). Quantification was determined at “baseline” (48 hrs after CTB injections), and at weekly intervals following ONT or sham ONT surgery for 4-weeks.

### Optic Nerve Transection

Surgical optic nerve transection (ONT) was performed unilaterally (on the right eye) using blunt dissection via a superior sub-Tenon's approach as previously described in detail [42]. The optic nerve was transected approximately 1.0 mm behind the globe and the ocular blood supply (ophthalmic artery) along the inferior aspect of the optic nerve was spared in all cases, as confirmed by CSLO video [40] (Spectralis HRA) immediately after completion of the procedure. In the sham-operated group, the surgical procedure was identical, up to and including the optic nerve sheath incision; however, in these animals the optic nerve was not transected. In all cases, antibiotic combination ointment (neomycin, polymyxin B sulfates and dexamethasone, Falcon Pharmaceuticals Ltd, Fort Worth, Texas) was applied topically and buprenorphine analgesic (0.075 mg/kg, IM) was administered after surgery and again 16–24 hrs later.

### Immunohistochemistry

Animals were injected with an overdose of pentobarbital (Euthasol, 0.7–1.4 ml/kg IP, Virbac Animal Health Inc., Fort Worth, Texas). Under a state of deep anesthesia, both eyes were enucleated, then death and tissue fixation occurred by transcardial perfusion of 125–150 ml of cold 4% paraformaldehyde in 0.5 M phosphate buffer (PB, pH 7.35). The retina was dissected from each globe under 4% paraformaldehyde in 0.5 M phosphate buffer, then flat-mounted for immediate fluorescence microscopy (for a second determination of CTB-labeled RGC density) and for subsequent immunohistochemical evaluation of astrocyte and microglia morphology and density. Retinas were rinsed three times for 15 min each in phosphate buffered saline (PBS, pH 7.4), treated ≥4 hours with Triton-X (3%) at 4°C, rinsed an additional three times in PBS, then pre-incubated overnight at 4°C in goat blocking serum (5%, diluted in PBS). Retinas were then transferred to new blocking buffer containing primary antibodies for 5 days at 4°C. Astrocytes were labeled using a primary antibody for the marker glial fibrillar acidic protein (GFAP, mouse monoclonal, 1∶400, Sigma-Aldrich Inc., St. Louis, MO, #G3893). Microglia were labeled using a primary antibody for the marker Iba-1 (1∶500, rabbit, polyclonal, Wako Pure Chemical Industries Ltd., Richmond VA, #019-19741). All retinas with CTB already present (Groups 1 and 3) were subsequently single-labeled with rabbit anti-Iba-1, whereas the retinas without CTB (Group 2) were double-labeled with both mouse anti-GFAP and rabbit anti-Iba-1. Secondary antibodies were goat anti-rabbit IgG coupled to AlexaFluor 594 diluted 1∶400 in 5% goat blocking serum, and goat anti-mouse IgG coupled to AlexaFluor 488 diluted 1∶400 in 5% goat blocking serum (Molecular Probes or Invitrogen).

### *Ex Vivo* Quantification of CTB-labeled RGC and Microglial Cell Densities

To quantify the density of fluorescent (CTB labeled) RGCs in whole-mount retinae, micrographs centered on the optic disc were acquired using a digital camera (Retiga 1300, QImaging, Surrey, BC, Canada) mounted to a DMRXE microscope (Leica Microsystems, Wetzlar, Germany) with a 5× air objective and filter set #513808 (450–490 nm excitation, 515 nm long pass emission, Chroma Technology Corporation, Bellows Falls, VT, USA). The size of each micrograph was 5.93 mm^2^ and the area of retina quantified was 5.8±0.1 mm^2^ (after excluding the optic disc area). RGC counts and density were obtained using ImageJ in the same manner as done for CSLO images.

All retinas that were stained with anti-Iba-1 were imaged using the same microscope and camera but at higher magnification (20× air objective) with filter set #513812 (515–560 excitation/590 long pass emission, Chroma). The location sampled in each retina was adjacent to the location of optic disc in the superior and inferior quadrant and matched between the two eyes of each animal. Manual focus was used to capture four images at each location corresponding to the four clearly distinct layers of tiled microglia (within the RNFL/RGC layer, the inner plexiform layer, the inner nuclear/outer plexiform layer and a very sparse array within the distal retina amongst the photoreceptor outer segments, approximately one-tenth the density observed in the proximal retinal layers). Each micrograph was converted to a 16-bit image in ImageJ, a Gaussian blur filter was applied and the background was subtracted to obtain microglia counts and density.

Confocal microscopy was used to evaluate three-dimensional colocalization of CTB and Iba-1 fluorescence and search for instances where microglia might have incorporated the RGC label as has been previously reported [16]. Confocal images were acquired in all retinas using a Leica DM RXE/TCS SPL microscope and 20× objective (PL APO; NA = 0.70). Laser lines at 488 nm and 633 nm were used to excite AlexaFluor 488 and AlexaFluor 594, respectively, spectral emission bands were set to 550–580 nm and 650–750 nm, respectively. Image dimensions were 750×750 µm (1024×1024 pixels, 0.732 µm pixel size, 0.60 µm voxel depth) and two frames were averaged for each axial slice. Typically 100–110 axial sections were required to encompass signal present throughout the depth of each retina (inter-quartile range 89–116).

### Statistics

Longitudinal changes in SD-OCT measurements of RNFLT and CSLO measurements of RGC density for each group were assessed using raw data and the generalized estimating equation (GEE), a method that takes into account correlation between the two eyes of each subject. Paired student t-tests with Bonferroni correction were applied to evaluate differences between time points within each group. Ordinary least squares linear regression and Deming linear regression were applied to assess the relationship between RGC densities measured *in vivo* by CSLO and *post mortem* by whole-mount retinal microscopy, as well as to assess the relationship between RGC density measured *in vivo* and RNFLT. A one-way ANOVA with Bonferroni post-hoc test was applied to compare microglial (Iba-1 positive) cell densities across experimental groups.

## Results

**Figure 1:**
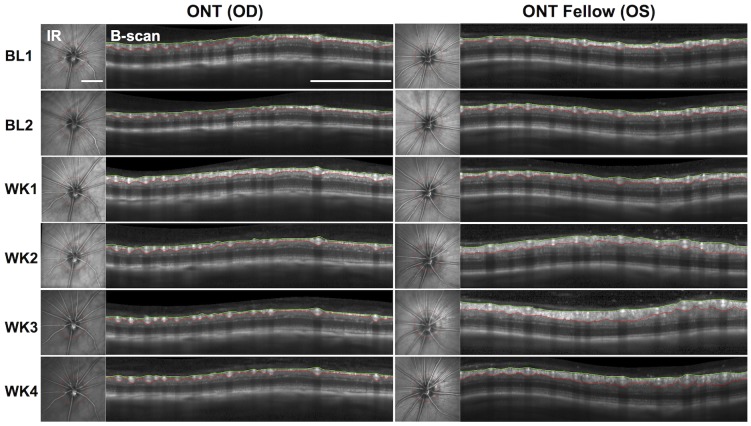
Representative individual example showing results for longitudinal SD-OCT measurements of RNFLT over time, beginning at baseline (BL) and continuing weekly for 4-weeks (WK1-WK4) after unilateral optic nerve transection (ONT) in the right eye (OD) and the non-operated fellow eye (OS). In each panel, the infrared (IR) reflectance image of the ocular fundus is shown at the left and the accompanying SDOCT B-scan is shown at the right; the red circle in the IR image shows the position and path of peripapillary SD-OCT scan. The B-scan images also show the segmentations used to derive RNFLT measurements: green, internal limiting membrane (ILM); red, posterior border of RNFL. RNFLT decreased over time in the ONT eye, as expected, but increased substantially over time in fellow eye. Scale bars = 1 mm.

**Figure 2:**
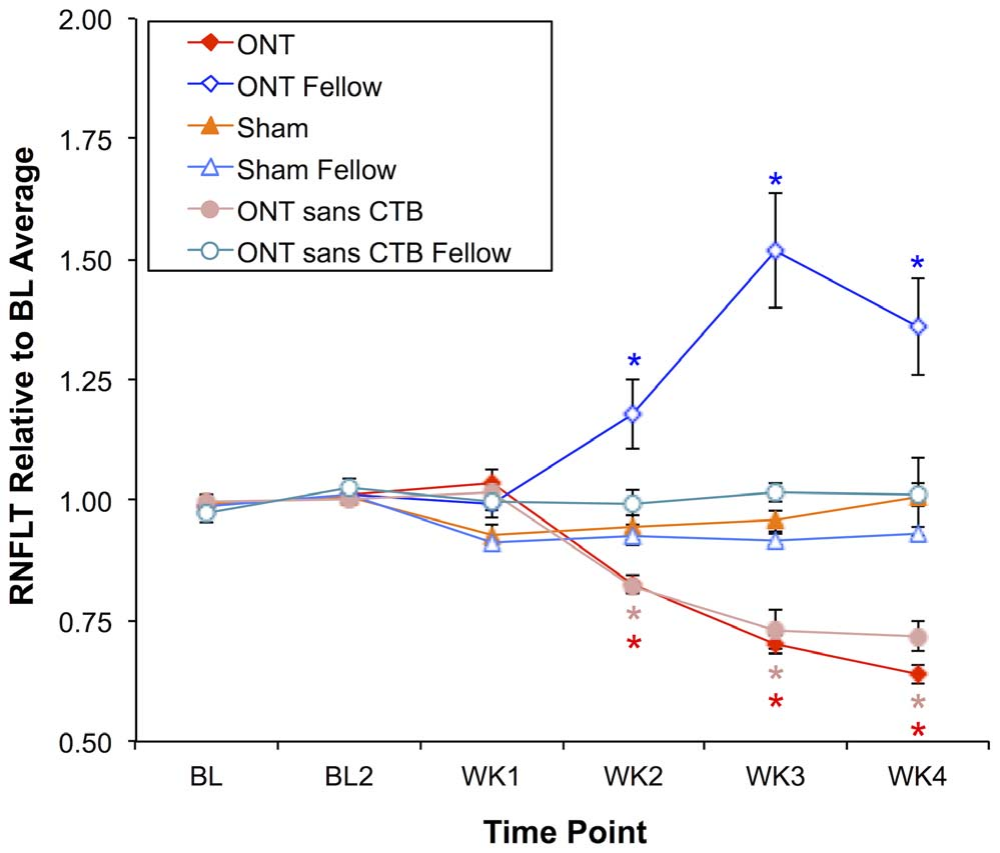
Longitudinal measurements of RNFLT for all three experimental groups. Values of RNFLT were normalized to the baseline average of each eye; symbols represent the group average, error bars indicate SEM. RNFLT decreased in Group-1 ONT eyes by 17%, 30% and 36% at weeks 2, 3 and 4. Group 2 operated eyes (“ONT sans CTB”) exhibited a similar pattern of longitudinal RNFLT change. In Group-1 ONT *fellow eyes*, RNFLT *increased* by 18%, 52% and 36% at weeks 2, 3 and 4. There were no significant differences from baseline for any group at week-1. There was no significant change in RNFLT at any follow-up time point in Group-2 non-operated fellow control eyes (“ONT sans CTB Fellow”), Group-3 sham operated (“Sham”) or Group-3 fellow control eyes (“Sham Fellow”). *Significant change from baseline assessed by two-way repeated measures ANOVA with Bonferroni post-hoc tests on raw values (see text for details).

Figure 1 shows the results in a single representative animal of RNFLT measured longitudinally by SD-OCT before and after unilateral ONT. As expected, RNFLT decreased steadily in the weeks after ONT (left column), however somewhat surprisingly, there was a substantial increase in RNFLT in the fellow eye during the second, third and fourth weeks of post-operative follow-up (right column). In this individual, there was also a small increase in RNFLT as well as an increase in the apparent intensity of RNFL reflectivity1-week after ONT, a finding that was not consistent enough across Group-1 ONT eyes to be statistically significant (group average change at week-1 was 3.5% increase over baseline average, p>0.05, see Fig. 2).

Figure 2 shows the aggregate results for RNFLT over time in each experimental group. In Group-1 animals, the longitudinal course of RNFLT changes differed significantly between the ONT-operated eyes and their fellow, non-operated eyes (p<0.0001, GEE interaction terms, eye/treatment versus time at weeks 2, 3 and 4). RNFLT decreased significantly in the ONT eyes of Group-1 animals by 17% (p<0.05), 30% (p<0.0001), and 36% (p<0.0001) relative to baseline at weeks 2, 3, and 4, respectively, but was not significantly different from baseline at week-1 (p>0.05). Interestingly, a substantial *increase* in RNFLT was observed in Group-1 fellow eyes, whereby it increased by 18%, 52% and 36% at weeks 2, 3, and 4, respectively (p<0.0001 each).

In contrast, among Group-2 animals only the ONT eyes exhibited significant change over time (p<0.0001, GEE interaction terms, eye/treatment versus time at weeks 2, 3 and 4), decreasing in the same manner as the ONT eyes of Group-1 animals. RNFLT decreased by 18% (p<0.001), 27% (p<0.0001), and 28% (p<0.0001) at weeks 2, 3, and 4, respectively in the ONT eyes, but did not change significantly in the non-operated fellow eyes at any follow up time point (p>0.05). This suggests that the remarkable fellow eye effect observed in the Group-1 animals is due to some interaction between the optic nerve injury to the contralateral eye and the presence of the axonal transport tracer CTB in both eyes. This is further supported by the results for RNFLT in the Group-3 animals, which are also shown in Fig. 2 and demonstrate that no significant change occurred in either the eyes that underwent ONT sham surgery or their fellow eyes, despite having had bilateral stereotactic injections of CTB and RGC labeling by retrograde transport (p = 0.71, GEE, eye/treatment effect; p = 0.58, 0.45, 0.08, 0.32, interaction terms, eye/treatment versus time at weeks 1, 2, 3 and 4 of follow-up, respectively). Thus, neither the presence of CTB alone, nor ONT of the contralateral eye alone were sufficient to cause the dramatic effect observed in the fellow eyes of Group-1 animals.

The analysis of retinal thickness changes distal to the RNFL (i.e. based on the values derived as total RT minus RNFLT, data not shown) revealed a small but significant degree of thinning in the Group-1 ONT eyes at week-4 (4.0% thinner than the baseline average, p<0.05) and an equally small but significant *increase* in thickness in the non-operated Group-1 fellow eyes at week-3 (5.5% thicker than baseline average, p<0.0001). The distal retinal thickness in the ONT-operated eyes of Group-2 animals was also slightly thinner than baseline during week-2 (by 3.8%, p<0.01), week-3 (by 2.7%, p<0.05) and week-4 (by 4.0%, p<0.001) of follow-up, but there were no significant changes for distal retinal thickness in their fellow eyes. There were no significant changes from baseline in the Group-3 sham-operated or fellow eyes, though there was a trend toward a small amount of thinning on average (2.1±2.9% below baseline).

**Figure 3:**
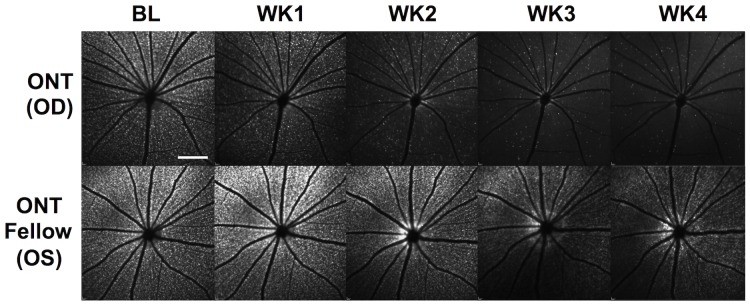
Representative individual example of longitudinal CSLO imaging *in vivo* (same animal as shown in Fig. 1) to assess the change in CTB-labeled RGC density over time after ONT in the right eye (OD) and in the non-operated fellow eye (OS). RGC density in the ONT eye decreased rapidly; RGC density in the fellow eye remained relatively constant, though decreased slightly over time due to fading of CTB fluorescence. Scale bar = 2 mm.

Figure 3 shows a representative individual example of the change over time in CTB-labeled RGC density after unilateral ONT in the right eye (OD) and in the non-operated fellow eye (left eye, OS) of a Group-1 animal (same animal as shown in Fig. 1). Though the apparent intensity of CTB label fades in the non-operated fellow eye (bottom row) over the course of 4-weeks follow-up (with camera sensitivity fixed over time), the ONT eye clearly exhibits a faster rate of decline.

**Figure 4:**
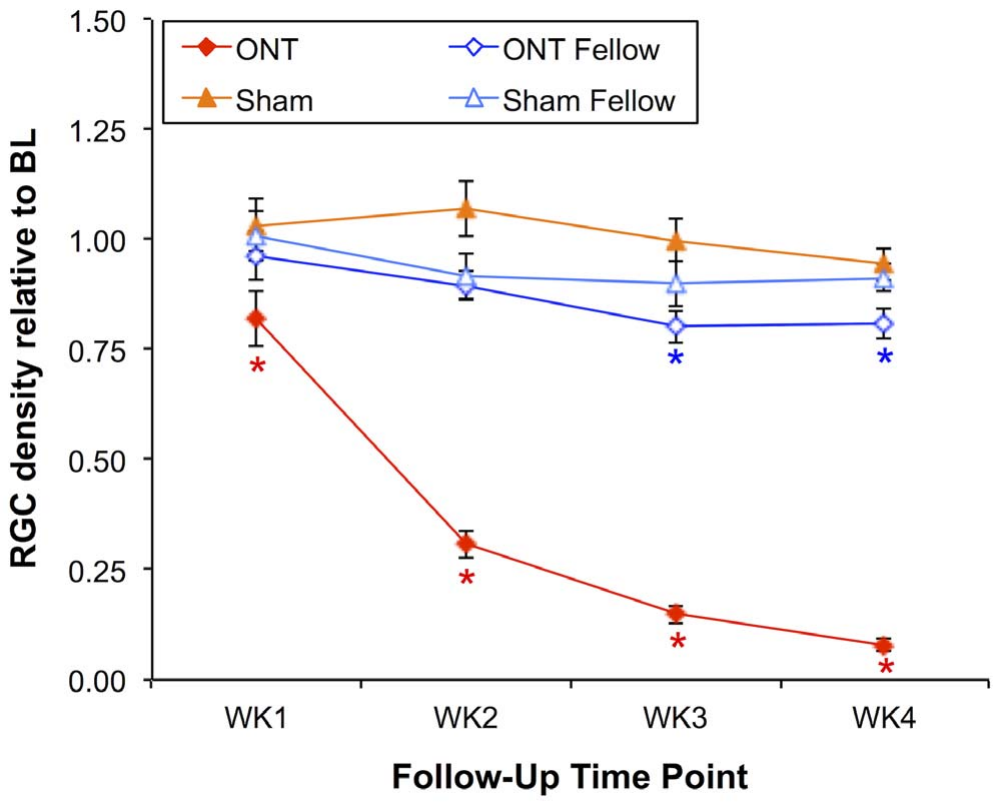
Longitudinal measurements of RGC density *in vivo* by CSLO in Group-1 (ONT versus ONT fellow eyes) and Group-3 (sham ONT versus sham fellow eyes) animals. The density of CTB-labeled RGCs decreased in ONT eyes by 18%, 69%, 85% and 92% relative to baseline at follow-up weeks 1, 2, 3 and 4, respectively. RGC density significantly decreased at week-3 by 20% and decreased 19% by week-4. RGC density did not change significantly in sham or sham-fellow eyes. *Significant change from baseline assessed by two-way repeated measures ANOVA with Bonferroni post-hoc tests on raw values. Error bars indicate SEM.

The aggregate results for both Group-1 and Group-3 animals (which had CTB labeling of RGCs) are shown in Figure 4. RGC density decreased in ONT eyes by 18%, 69%, 85%, and 92% relative to baseline at weeks 1, 2, 3 and 4 (p<0.0001 each), but were also significantly reduced at week-3 (by 20%; p<0.0001) and week-4 (by 19%; p<0.001) in the Group-1 fellow eyes (p = 0.001, GEE, eye/treatment effect; p<0.01 at week 1 and p<0.0001 at weeks 2, 3 and 4, GEE interaction terms, eye/treatment versus time). In Group-3, there was no significant difference between sham-ONT operated eyes and their fellow eyes (p = 0.15, GEE), no significant interaction with treatment during follow-up (p>0.10, GEE) and no significant difference from baseline at any follow-up time point in either group.

**Figure 5:**
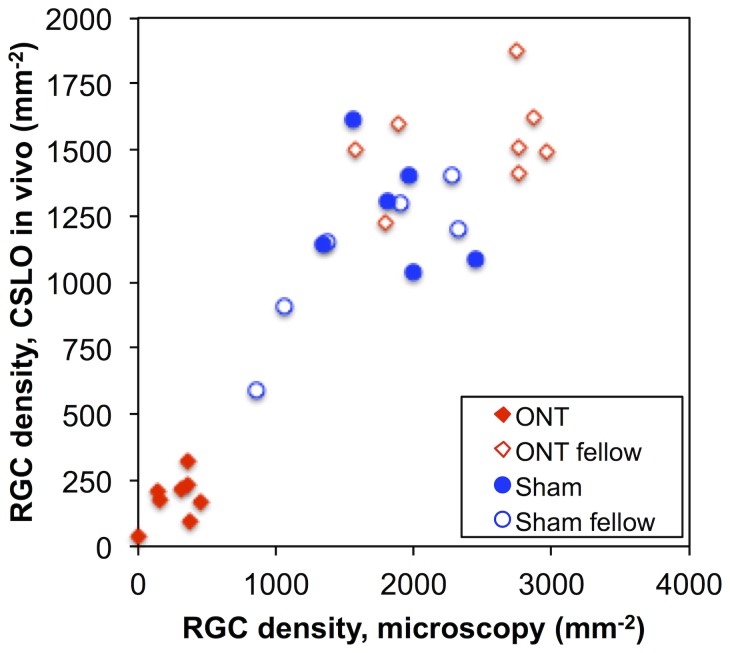
The relationship between RGC density measured *in vivo* by CSLO and RGC density measured *post mortem* by epifluorescence microscopy at week-4.

The data plotted in Figure 5 demonstrate that the RGC density derived *in vivo* from CSLO images acquired during the final follow-up time point (week-4) were well correlated with RGC density measurements derived ex vivo from epifluorescence microscopy images of flat-mount retinae (Pearson R = 0.91, p<0.0001). Since both estimates contain inherent error, Deming regression was applied to determine the slope and intercept of the relationship, which resulted in the following equation: RGC density by CSLO *in vivo* (per mm^2^) = 0.58*RGC density by microscopy ex vivo (per mm^2^)+103 (per mm^2^), similar to the findings in two of our previous studies [26], [39]. There is evidence in these data of “saturation” whereby a quadratic model provided a statistically better fit than a linear model (F = 13, p = 0.001), suggesting that the resolution of the *in vivo* CSLO method places an upper limit on density measurements. Thus the limitations of imaging *in vivo* (lower gain, contrast and resolution) result in an underestimate of RGC density as compared with *post mortem* microscopy, but there is a strong correlation between the two over most of their dynamic ranges. Note that one or two retinas from the Group-3 (sham) fellow eyes had lower densities despite adequate post mortem assessment of CTB fluorescence at the injection site in the superior colliculus. Their results are presumed to be due to limited uptake and/or transport of CTB from the superior colliculus and although they may be “outliers”, they do not affect the correlation between *in vivo* CSLO and ex vivo microscopy counts.

**Figure 6:**
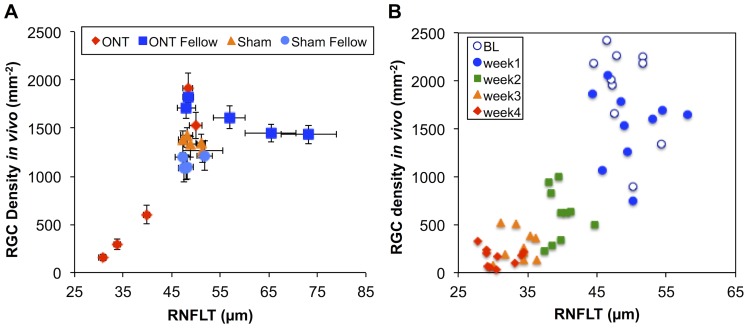
The relationship between RGC density measured *in vivo* by CSLO and RNFLT measured *in vivo* by SD-OCT. Longitudinal data for both groups that had RGCs labeled by CTB (Groups 1 and 3) are plotted together in panel A; error bars indicate SEM. In panel B, only the longitudinal data for the individual ONT eyes are plotted.

The original motivation and purpose of this study was to evaluate the relationship between longitudinal measurements of RNFLT and RGC density *in vivo*. Figure 6A presents this comparison for both groups that had RGCs labeled by CTB (Groups 1 and 3). The apparent relationship is not readily described by a simple function. The data for the sham-ONT and their fellow eyes form a cluster around an average RNFLT of 49 µm and RGC density of 1250 per mm^2^. This reflects the fact that these eyes did not exhibit any significant longitudinal change, so their data do not contribute much toward revealing the relationship of these parameters beyond serving as an anchor around the ‘normal’ state. One aspect of the complicated relationship shown in Fig. 6A is that the Group-1 fellow eyes (blue squares) exhibited a dramatic increase in RNFLT during weeks 2–4 after the contralateral eyes underwent ONT (Figs. 1–2), yet their RGC density did not change significantly (Figs. 3–4). Another aspect of the complicated relationship depicted in Fig. 6A is due to the fact that the ONT eyes of Group-1 exhibited a rapid decline in RGC density from baseline to week-1 after ONT (from 1917 to 1527 mm^−2^), yet there was no significant change in RNFLT during that same span.

Since the dramatic increase in RNFLT from Group-1 fellow eyes was such a strong complicating factor, the comparison between these two parameters was limited to just the Group-1 ONT eyes as shown in Figure 6B. The severity of the optic nerve injury in these eyes led to substantial changes in both parameters, enabling comparison over a wide range of observations. For the data plotted in Fig. 6B, the correlation between RGC density measured *in vivo* and RNFLT was 0.81 (Pearson R, p<0.0001). Despite the tendency for a RGC density to change more rapidly than RNFLT in the first week after ONT, a linear model proved to be a superior description of these data than a higher-order (quadratic) model (F = 0.6, p = 0.43). Therefore Deming regression was applied to determine the slope of this linear relationship, which resulted in the following equation: RGC density measured by CSLO *in vivo* (mm^−2^) = 114*RNFLT (µm) – 3742 (mm^−2^). We further carried out the same analyses by quadrant and found again that a linear model provided a statistically superior fit for the data of each quadrant as compared with a quadratic model (F = 0.5, 0.2, 1.8, 0.2; p = 0.50, 0.67, 0.18, 0.68, respectively, for the temporal, superior, nasal and inferior quadrants). There were no differences by quadrant for the best-fit linear function relating RNFLT to RGC density (F = 1.5, p = 0.20) as might be predicted for a complete axotomy injury model. The average Pearson correlation coefficient was 0.76 (range 0.68 to 0.82, p<0.0001 for each), suggesting the good relationship should exist for models with more localized damage.

**Figure 7:**
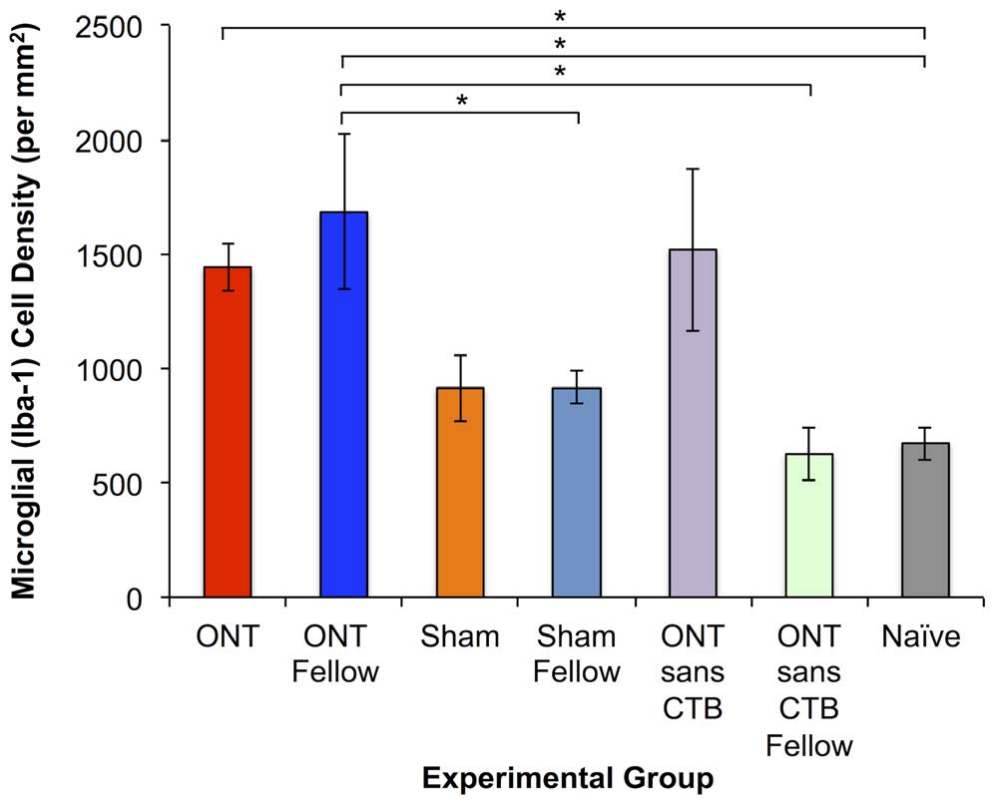
Density of Iba-1 positive (Iba-1+) retinal microglial cells in each of the experimental groups as well as in a group of naïve control eyes. Microglial cell density was elevated 4 weeks after ONT, whether CTB was present or not, but also substantially elevated in ONT-fellow eyes only if CTB was present. *One-way ANOVA with Bonferroni post-hoc tests (p<0.05). Error bars indicate SEM.

In order to determine whether there was evidence that the dramatic increase in RNFLT observed in the fellow eyes of Group-1 ONT animals was specifically associated with activation of retinal glia, *post mortem* evaluation of whole-mount retinae was carried out using immunohistochemical markers of Iba-1 (microglia) and GFAP (astrocytes ± Müller glia). Figure 7 shows the density of Iba-1 positive (microglial) cells observed in each group of eyes. Note, the data shown in Fig. 7 represent the total two-dimensional density (total sum count per mm^2^) of four distinctly stratified populations of Iba-1 positive cells through the depth of the retina; though the absolute density varied by layer, the pattern of results (relative differences between groups of eyes) was similar for each individual stratum (data not shown). In naïve eyes, microglial cell densities in each of the four individual layers (proximal to distal) were: 253, 227, 171 and 22 per mm^2^.

Figure 7 shows that there is a significant increase in the density of retinal microglia in all eyes that had an ONT 4-weeks earlier (Group-1 ONT eyes, 2.2× increase over naïve control eyes; Group-2 ONT eyes without CTB label of RGCs, 2.3× increase over naïve control eyes, p<0.05 each). This was specific since there was no significant difference observed between sham-ONT (Group-3) operated eyes and naïve eyes. However, Figure 7 also shows that there was a significant increase in retinal microglial cell density in Group-1 ONT fellow eyes (2.5× increase over naïve control eyes, p<0.05), which was specific to that group since the Group-3 ONT fellow eyes (that did not have CTB labeling of RGCs) did not exhibit any significant increase as compared to naïve eyes. Moreover, the density observed in the fellow eyes of Group-1 ONT animals was significantly greater than both of the other groups of fellow eyes (p<0.05), which themselves were not significantly different from naïve eyes.

**Figure 8:**
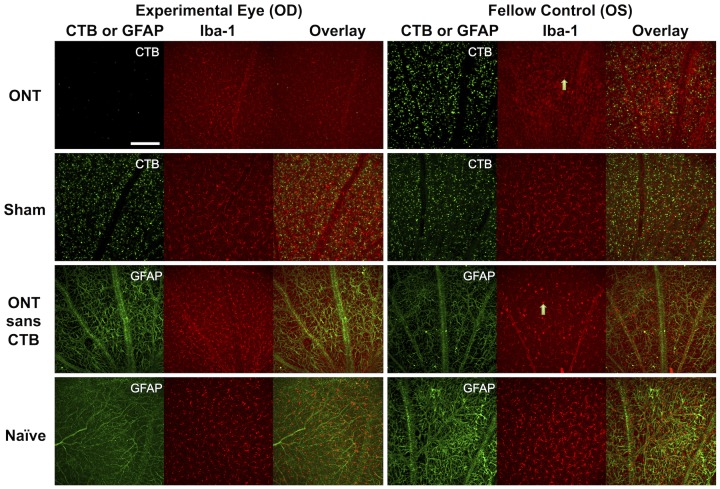
Representative images from each group of experimental eyes, fellow control eyes and naïve control eyes acquired by confocal microscopy. Increased microglial (Iba-1 positive) cell density was evident in Group-1 ONT eyes, their fellow eyes and in the Group-2 ONT eyes (sans CTB labeling of RGCs). The majority of microglia within the anterior most plexus exhibited morphological characteristics consistent with the ‘activated’ state, including enlarged, rounded or ameboid soma and retracted processes. See text for additional details (arrows). Scale bar = 0.19 mm.

In contrast to the results shown in Fig. 7 for retinal microglia, there were no consistent changes observed in retinas stained with an anti-GFAP marker. Figure 8 shows representative images of retinal flat-mounts acquired by confocal microscopy in each of the experimental groups. Increased microglial (Iba-1 positive) cell density was evident in Group-1 ONT eyes, Group-1 ONT fellow eyes, and Group-2 ONT eyes that did not have RGC labeling by CTB. Microglia in both groups of ONT-fellow eyes also exhibited signs of being in an activated state (such as altered morphology: larger size, rounder or ameboid in shape, with fewer, thicker processes; arrows), particularly within the RNFL/RGC layer. However, consistent with the results of quantitative density analysis shown in Fig. 7, the most impressive subjective indications of microglial activation were observed in the fellow eyes of Group-1 animals (i.e. those in which there was a combination of ONT to the contralateral eye and bilateral CTB labeling of RGCs). Microglial density and morphology in Group-3 sham-ONT operated eyes and their fellow eyes were both comparable to naïve eyes. There were no changes in astrocyte (GFAP-positive) appearances between naïve and Group-2 eyes (without CTB labeling of RGCs, either in the ONT eye or their fellow eyes). Colocalization of CTB fluorescence and Iba-1 staining was not observed in any of the ONT, ONT-fellow, sham or sham-fellow eyes, which indicates that active microglial phagocytosis of CTB was nearing completion by the 4-week time point.

## Discussion

The primary goal of this study was to evaluate the relationship between measurements of RGC density obtained *in vivo* by CSLO directly with measurements of RNFLT obtained *in vivo* by SD-OCT. We found that there was, in general, a strong correlation between these two parameters (Pearson R = 0.81). Though this relationship has not previously been reported, this result was anticipated since each parameter has been previously shown to decline after optic nerve injury [10]–[14], [17], [18], [20], [25], [35], [36]. RNFLT is also known to correlate well with optic nerve axon counts [43], which should in turn reflect the number of surviving RGCs [44]. However, the relationship between *in vivo* measurements of RGC density and RNFLT was complicated by three factors.

The first of these factors is that the CSLO measurement of RGC density underestimates by approximately one-third the density measured *post mortem* by microscopy of flat-mount retinae (Fig. 5 and our previous studies [26], [39]). This in itself should not affect the slope or strength of the relationship between RGC density and RNFLT, however, the data in Fig. 5 also suggests that the underestimate increases for the highest densities (i.e. nonlinear), presumably because the limitations of imaging *in vivo* (lower contrast and resolution) impose an upper limit on the measurable density which is lower than that achievable by *post mortem* microscopy. Despite this caveat, the results demonstrate that RNFLT should represent RGC density over most of the important portion of the range where the effects of injury can be followed longitudinally. It is likely that the addition of adaptive optics to compensate for the higher-order aberrations of the eye would increase the dynamic range available for measurements of RGC density acquired by *in vivo* imaging [22]–[24].

The second complicating factor we observed in this study was that the decline in RGC density had a more rapid onset after ONT than did the decline in RNFLT, which was delayed by ∼1 week (compare Figs. 2 and 4 and note nonlinear trend in Fig. 6B). It is unclear whether this discrepancy represents some aspect of RNFLT thickening 1 week after ONT that might be offsetting axon thinning or loss (recall we did observe a small, non-significant 3.5% increase in RNFLT at week-1 follow-up). For example, it is possible that degenerative axon changes such as bulb and spheroid formation and sprouting could result in either an increase of RNFLT or an offset of thinning associated with loss of other axons [45]. Early infiltration and activation of microglia might also offset RNFL thinning due to axon degeneration [16]. We have recently reported a transient increase in RNFLT that peaks 3–7 days after an 8-hour duration of acute IOP elevation to 50 mmHg [39]. The underlying cause of that more transient phenomenon might also be operative during the first week (or longer) after ONT. It is also possible that the RGC label used in this study (CTB by retrograde transport) and/or the RGC somas disappear after ONT more rapidly than do their axons from the RNFL, consistent with a previous report [46]. However, there is a substantial body of evidence that the opposite phenomenon occurs, both in response to axotomy [16] and in experimental models of glaucoma based on chronic intraocular pressure elevation in rodents [47]–[55]. Similarly, using CSLO to perform longitudinal imaging *in vivo*, Leung et al [21] have shown numerous examples of RGC somas persisting longer than their axons after calibrated optic nerve crush injury, though the time course of all changes after crush injury is generally slower than that after ONT. The study by Leung et al [21] also demonstrated clearly that dendritic tree shrinkage was consistently the earliest morphological change detectable by *in vivo* imaging after crush injury, which likely explains the relatively subtle loss of retinal thickness distal to the RNFL observed in the ONT eyes of our study.

The third complication we observed for the overall relationship between RNFLT and RGC density was due to the unexpected dramatic increase of RNFLT in the fellow eyes of Group-1 ONT animals (Figs. 2 and 6A). This phenomenon peaked 3 weeks after ONT when there was otherwise a 20% reduction in RGC density measured by CSLO. Results of the control studies (Groups-2 and 3) demonstrate clearly that this effect is peculiar to only those fellow eyes of ONT-operated animals that had their RGCs labeled bilaterally by retrograde transport of CTB. Neither ONT alone (without CTB label, Group-2) nor the presence of CTB alone (sham ONT surgery, Group 3) resulted in any such increase of RNFLT. This phenomenon was also associated with a dramatic increase of retinal microglial cell density, which was most prominent within the RNFL/RGC layer (though also present in deeper retinal layers). The majority of microglia within the anterior most plexus exhibited morphological characteristics consistent with the ‘activated’ state, including enlarged, rounded or ameboid soma and retracted processes [56], [57]. The Group-1 ONT fellow eyes were also the only fellow eye group to manifest an increased microglial density (Fig. 7), matching the specific increase RNFLT in that group. This suggests microglial activation within the RNFL may be contributing to the RNFLT increase, or at least reflecting a common causative mechanism that is somehow triggered by an interaction between the severe injury to the contralateral eye and the presence of CTB tracer to label RGCs. Our recent observation of transiently increased RNFLT peaking 3–7 days after an 8-hour period of acute intraocular pressure elevation to 50 mmHg was not associated with increased microglial density (or RGC loss) [39], suggesting there may be important differences between these reactions. Further studies will be required to determine the precise nature of these phenomena.

It is well documented that microglial activation occurs in the retina very early after optic nerve injury, including experimental glaucoma, and persists for at least 4 weeks [13], [16], [57]–[60]. Thus it was no surprise to find increased microglial cell density in ONT eyes 4-weeks after injury (Fig. 7). Previous studies have also demonstrated a prominent fellow-eye effect, particularly with regard to microglial activation, following optic nerve injury [61]–[63] and experimental glaucoma [64], [65]. Interestingly, in the study by Sobrado-Calvo et al, the authors also reported evidence of an interaction effect between ipsilateral eye puncture and contralateral optic nerve injury [63]. Another recent report by Kezic et al [66] demonstrated that ipsilateral eye puncture (used as a control) and contralateral acute intraocular pressure elevation resulted in bilateral microglial activation. Our findings are generally consistent with these previous reports in that we observed only relatively subtle evidence of microglial activation in the fellow eyes of Group-2 animals 4-weeks after ONT in their contralateral eyes, consisting only of morphological signs without any significant increase in density at this relatively late stage. The fellow eyes of Group-1 animals in contrast, exhibit dramatic evidence of an interaction effect including both increased RNFLT and profound increase in microglial cell density that is peculiar to this group. As stated by these previous investigators, the fellow eye should not be the sole control group in unilateral models of optic nerve injury and experimental glaucoma [61]–[63], [65], [66]. Important additional controls should be included, such as a group of animals where the sham procedure or manipulation is made unilaterally, with the fellow eye operating as one level of control and ideally also another group of naïve animals to serve as comparison for sham and sham-fellow eyes. Our results stress further that this consideration will become increasingly important for experiments in which multiple manipulations are included, such as introduction of vital dyes or therapeutic agents (and vehicle controls) bilaterally before or after unilateral experimental injury. Future studies will aim to address the source of the interaction and intriguing contralateral eye effects.

Finally, it should be noted that the image segmentation used in this study to determine RNFLT included the major blood vessel profiles, which results in an offset above zero for the lower limit of the dynamic range. Measurements of RNFLT made between the vessels, such as done in a study by Nagata et al [35], would remove this relatively constant offset and result in a lower limit closer to zero (instead of the 36% loss versus baseline determined to be the dynamic range in this study).

In summary, the results of this study demonstrate that there is a strong correlation between longitudinal *in vivo* measurements of RNFLT and RGC density during the 4-week follow-up period after ONT. The results also revealed a strong fellow eye effect specific to the ONT plus CTB-labeled group (Group-1), which consisted of a dramatic increase in RNFLT associated with retinal microgliosis.
